# Dr. Charles P. Alling

**Published:** 1908-06

**Authors:** 


Dr. Charles P. Alling, of Dunkirk, died at his home May 8, 1908, aged 70 years. He graduated in medicine at the Cleveland Homeopathic Hospital College in 1862, and was a member of the state homeopathic state medical societies of New York and Ohio. He is survived by his wife and two children, Howard W. Alling and Mrs. Mary E. Fenger, the latter residing at Jamestown.

